# Simulated airplane headache: a proxy towards identification of underlying mechanisms

**DOI:** 10.1186/s10194-017-0724-3

**Published:** 2017-01-28

**Authors:** Sebastian Bao Dinh Bui, Torben Petersen, Jeppe Nørgaard Poulsen, Parisa Gazerani

**Affiliations:** SMI®, Department of Health Science and Technology, Faculty of Medicine, Aalborg University, Aalborg, Denmark

**Keywords:** Simulated airplane flight, Pressure chamber, Airplane headache, Cortisol, Prostaglandin E_2_, Biomarker, Pulse, Blood pressure, Thermo imagining, Saturation pulse oxygen

## Abstract

**Background:**

Airplane Headache (AH) occurs during flights and often appears as an intense, short lasting headache during take-off or landing. Reports are limited on pathological mechanisms underlying the occurrence of this headache. Proper diagnosis and treatments would benefit from identification of potential pathways involved in AH pathogenesis. This study aimed at providing a simulated airplane headache condition as a proxy towards identification of its underlying mechanisms.

**Methods:**

Fourteen participants including 7 volunteers suffering from AH and 7 healthy matched controls were recruited after meeting the diagnostic and safety criteria based on an approved study protocol. Simulation of AH was achieved by entering a pressure chamber with similar characteristics of an airplane flight. Selected potential biomarkers including salivary prostaglandin E_2_ (PGE_2_), cortisol, facial thermo-images, blood pressure, pulse, and saturation pulse oxygen (SPO) were defined and values were collected before, during and after flight simulation in the pressure chamber. Salivary samples were analyzed with ELISA techniques, while data analysis and statistical tests were handled with SPSS version 22.0.

**Results:**

All participants in the AH-group experienced a headache attack similar to AH experience during flight. The non-AH-group did not experience any headaches. Our data showed that the values for PGE_2_, cortisol and SPO were significantly different in the AH-group in comparison with the non-AH-group during the flight simulation in the pressure chamber.

**Conclusion:**

The pressure chamber proved useful not only to provoke AH-like attack but also to study potential biomarkers for AH in this study. PGE_2_, and cortisol levels together with SPO presented dysregulation during the simulated AH-attack in affected individuals compared with healthy controls. Based on these findings we propose to use pressure chamber as a model to induce AH, and thus assess new potential biomarkers for AH in future studies.

## Background

Airplane Headache (AH) occurs in a subset of general population during flights. It appears as an intense short lasting headache more frequently during take-off or landing [1–7]. The exact mechanism (s) underlying AH remains unclear; but, it has been suggested as a headache with a multifactorial pathogenesis. Current diagnosis is based on classification of headaches set by the International Headache Society (IHS) [1]. Some studies have attempted to reveal AH mechanisms through various tests including blood sampling and identification of potential abnormalities [8, 9]. Collectively, abnormal findings have not been detected in those who suffer from AH compared with those who do not [8, 9]. Perhaps it is because AH in nature is a short-lasting headache that is commonly terminated after the flight period is over and most of the investigations have been done outside the occurrence of the headache episode. Hence, further investigation with alternative methodology might prove suitable to study AH mechanisms. One assumption is that AH might be caused by a rapidly changing cabin-pressure, as it is seen during take-off and landing phases [2, 8, 10]. Shifting of cabin-pressure from low to high and reverse, is proposed to induce barotrauma within nasal sinuses or vasodilation in the cerebral arteries, which in turn can trigger AH [3, 10]. Currently, non-steroidal anti-inflammatory drugs (NSAIDs), such as ibuprofen or naproxen, are taken for AH, which suggests a possible contribution of an inflammatory state. Therefore, we have chosen to study some biomarkers in relation to pressure changes and development of transient inflammatory state and vasodilatory components that may occur in AH.

Hypobaric pressure is believed to trigger vasodilation due to a decrease in the atmospheric oxygen concentration that can cause a headache. This mechanism has been proposed for those individuals who suffer from High Altitude Headache (HAH) [11]. It has been suggested that HAH is a risk factor for AH [2]. HAH is defined by IHS as a headache that develops after ascent to altitudes above 2500 m and resolves within 24 h after descent to below 2500 m [1]. The pain intensity is mild or moderate and the headache is aggravated by e.g., movement, straining or bending. The characteristics of HAH imply that it is different from AH. It should be noted that AH also can occur at ascending phase. Even though HAH and AH are separate headaches, both headaches are classified under the same category;”10.1 Headache attributed to hypoxia and/or hypercapnia” by IHS [1]. It is known that hypoxia occurs during flight travels and elevated altitudes due to the changes in the atmospheric pressure [12, 13]. It is therefore interesting to examine if there is a potential relationship between these two types of headaches. If AH and HAH share a common mechanism, changes in cabin pressure might alter saturation pulse oxygen (SPO) and contribute in development of AH.

In addition, vascular input has been shown to play an important role in generation of headaches [14–18]. Vasomotor and blood flow changes due to cabin pressure changes during flight might trigger AH. One of the known vasodilators of cerebral arteries is prostaglandin E_2_ (PGE_2_) [14, 19]. PGE_2_ is produced in cerebral endothelial cells [20] and mast cells [21] and can be released by cabin pressure change; but, it is not known if this occurs in AH. However, PGE_2_ has been shown to contribute in the pathophysiology of neurovascular headaches [22]. Under experimental conditions, when PGE_2_ is infused, it also induces headache in healthy humans that is most likely due to activation and sensitization of sensory afferents innervating cranial vessels [14, 23].

Anxiety, fear and mental stress are not unusual phenomena among flight passengers. Previous studies have documented that mental stress is higher or occurs at a higher rate in passengers with AH-attacks [2, 9, 24]. Hence, it has been suggested that stress and anxiety may play a role in the development of AH [9]. This indicates that susceptible individuals with AH might even be at higher risk for developing stress before or during airplane travels. Flight phobia has also been suggested to increase mental stress during flight travels [24]. Cortisol, released at higher concentrations during stressful conditions, has been proposed to play a role in development of headaches including AH [6]. Studies have already shown an association between cortisol levels and other types of headaches [25]. However, the exact role of cortisol remains unclear in relation to AH and needs further investigation.

To test whether pressure changes would lead to alteration in serum oxygen and circulating PGE_2_ concentration, and if cortisol levels might be a factor in development of AH, this study was designed to simulate AH as a proxy towards identification of underlying mechanisms. Practical challenges for studying AH under real conditions led us to apply an alternative model to simulate flight-associated alterations in healthy individuals and those who have experienced AH by placing them in a pressure chamber under fully controlled conditions. It was proposed that pressure chamber would cause a headache with similar characteristics to AH and that it would be correlated with some alterations in some biomarkers such as alterations in blood perfusion, SPO, PGE_2_ and cortisol concentrations.

## Methods

### Study population

This AH-simulated study aimed at identification of mechanisms that might contribute in development of AH under a well-controlled environment. A pressure chamber (Training Chamber Low Pressure High Altitude Six Man Capacity, Model ATV-183, Aero-Test Equipment Co. Inc., Dallas, Texas, USA) was used as a proxy to simulate AH. The pressure chamber has been expanded to accommodate 7 participants instead of 6; hence, the number of recruited participants for each group was limited to 7. Two groups of AH-group and non-AH-group participated in this study. Participants were examined by the researchers and a medical authority in accordance to the AH criteria’s defined by IHS [1], in order to verify an accurate diagnosis of AH. The average age was 24 ± 1.6 years, where 4 of the participants were males and the remaining 10 were females. The study protocol was approved by the local ethics committee of Region Nordjylland (approval number N-20150026) and was conducted at the Center for Flight- and Naval Medicine, Skalstrup Airbase, Denmark. Written informed consent was obtained from all participants prior the study and travel expenses were compensated.

Those who were enrolled in the study were all adults above 18 years either healthy (non-AH-group) or with AH (based on the diagnostic criteria for AH according to IHS’ definition of AH [1]).

Individuals were excluded during screening, if they were pregnant, frequent users of any medications including paracetamol, triptans, having an addictive or previously addictive drug abuse, suffering from epilepsy, asthma, other primary headaches, migraine or any psychological and cardiovascular diseases. The applied criteria were maintained to ensure a high safety level for the participants and a controlled simulation of AH headache if occurred in the pressure chamber.

No participant was allowed in stepping the pressure chamber if a headache occurred in the trial day due to any reason. Participants were also asked not to eat or drink coffee, chili, nicotine, or chocolate and to avoid brushing teeth or using mouthwash prior to the study (see saliva assessments below). Participants were also asked to avoid demanding exercise or energy drinks that might had an influence on the study outcomes.

Participants were asked to rate their pain intensity on a scale of 1–10, where 1–3 was considered as mild headache, 4–7 reflected moderate headache, and 8–10 was considered as severe headache.

### Experimental setup: pressure chamber

Participants were instructed to sit in a pressure chamber, which was located in a military airbase, Skalstrup Airbase, Denmark. Two safety observers from the airbase were present during the entire procedure, ensuring that every steps proceeds safely. The chamber contained 7 seats. Participants seated in the pressure chamber for approximately one hour to experience a simulated airplane flight by altering the pressure and air composition similar to what occurs during a real flight [26]. The pressure switched from the atmospheric pressure observed at ground level, to the pressure value present at an altitude of 2438 m, and returned to the ground level when the time was over. The ascending and descending speed were set to 457.2 m/min ≈ 0.055 bar/min. The ascending and descending periods lasted for 5–6 min each, while the cruising phase lasted for almost 47 min, in an altitude of 2438 m to simulate closely what occurs during a one-hour flight in the real-world condition. No service was provided during simulation period and ear protection was provided for all participants to filter the noise. Eyes were kept open and no participant slept during the trial.

### Potential biomarker assessments

A selection of assessments was used at 3 allocated time points: before (pre-simulated flight), during (during simulated flight) and after (post-simulated flight) the exposure to the pressure chamber. Baseline assessments were conducted within 30 min prior to entrance to the pressure chamber. Second assessments were conducted in the middle of the simulated flight, when the participants had been present in the chamber for 30 min, where the maximal altitude and pressure had been achieved. The third and final assessments were conducted within 15 min after participants left the chamber. Saliva samples were collected in each allocated time point for ELISA-analysis of PGE_2_ and cortisol. Each sample consisted 1.5 ml natural saliva without any stimulation. The participants delivered the samples by continuously salivating in a small cup, hereafter the saliva samples were transferred to plastic centrifuge tubes by single use plastic pipettes. The total amount of collected saliva for each participant was 4.5 ml. All samples were placed in −80 °C freezer and were kept frozen until analysis.

Blood oxygen, pulse and blood pressure were assessed at the allocated time points. This allowed measurements of the participants’ pulse frequency, systolic and diastolic blood pressure as well as the SPO-concentration in their blood. Blood pressure and pulse were assessed by using a pressure cuff (BP-102 M, Hangzhou Sejoy Electronics & Instruments Co, Hanzhou, China), while the oxygen saturation was assessed by using a finger-based pulse oximeter (CMS50d, Contec Medical Systems Co, Qinhuangdao, China).

A portable compact thermo-camera (FLIR systems E60 thermal imager, FLIR Systems, Wilsonville Oregon, USA) was used to capture thermo-images of participants’ faces to record the average facial skin temperature at each dedicated time point. The thermo-camera was calibrated before use. The sensitivity of the device was <0.05 °C. Temperatures in the waiting room and the pressure chamber were recorded and environmental humidity was also monitored. All images were transported on a USB pen and stored in Excel 2010 (Microsoft Corp., Seattle, WA, USA) for further analysis.

All participants were asked to report the possible occurrence of headache and its characteristics before, during, and after their stay in the pressure chamber. They were all provided with a specific questionnaire for each time point, which aimed at documenting the findings, and ensuring the safety of our participants. The participants were asked to fill out details of their current and recent health situation, and were requested to note any changes that might occur during their stay. While in the chamber, participants were asked to continuously note and rate possible symptoms related to AH, which included pain intensity rating, duration, location of headache, type of headache as well as stress level and general wellbeing. These findings were then collected and compared with the international guidelines for AH in order to ensure matching the criteria set by IHS.

## Statistical analysis

Data handling was conducted in Excel 2010 (Microsoft Corp., Seattle, WA, USA). All statistical tests were conducted in SPSS version 22.0 (IBM Corp., Armonk, NY, USA). All graphical illustrations were created with GraphPad Prism version 7.00 (GraphPad Software, La Jolla, California, USA). Data normality was assessed by Shapiro-Wilk’s Test of Normality. A two-way repeated measures ANOVA was performed to determine the impact of simulated airplane headache on concentrations of PGE_2_, cortisol, oxygen saturation, pulse and blood pressure at dedicated time-points of pre-simulated flight, during the simulated flight and post-simulated flight. Level of significance was set at *p <* 0.05. Data are presented as mean ± SD (standard deviation).

## Results

### Incidence of headache during the simulated flight

Fourteen participants completed this study. Seven were admitted to the non-AH-group, as they did not have any experience with bouts of AH, while the remaining seven participants were placed in the AH-group, as they had frequently experienced bouts of AH while travelling by flight.

No participants in the non-AH-group developed a headache or any accompanying symptoms, and none were reported stress or anxiety while in the pressure chamber.

All seven participants of the AH-group experienced a headache with AH characteristics in accordance with IHS’ criteria. The majority of AH participants (43%) described the location of their headaches as fronto-orbital. The quality of the headache was described mainly as stabbing (43%). The pain intensity was described as severe (58%) or moderate (29%) (see Table 1).

**Table 1:** Demographic characteristics and clinical reports from the AH participants

Subject no	Gender	Age (year)	AH during simulated flight?	Duration of AH (minutes)	Intensity of pain	Quality of pain	Localization of the headache	Stress during simulated flight?	Anxiety during simulated flight?
1	F	22	Yes	15	4	Pulsating	Unilateral	No	Yes
2	F	29	Yes	20	10	Stabbing	Fronto-orbital	No	No
3	F	23	Yes	30	8	Stabbing	Unilateral	No	Yes
4	F	24	Yes	20	3	Jabbing	Fronto-parietal	No	No
5	F	25	Yes	40	7	Pulsating	Fronto-parietal	No	Yes
6	M	23	Yes	40	3	Stabbing	Fronto-orbital	No	No
7	F	51	Yes	20	8	Pulsating	Fronto-orbital	Yes	Yes

### Cortisol concentration

**Fig. 1:**
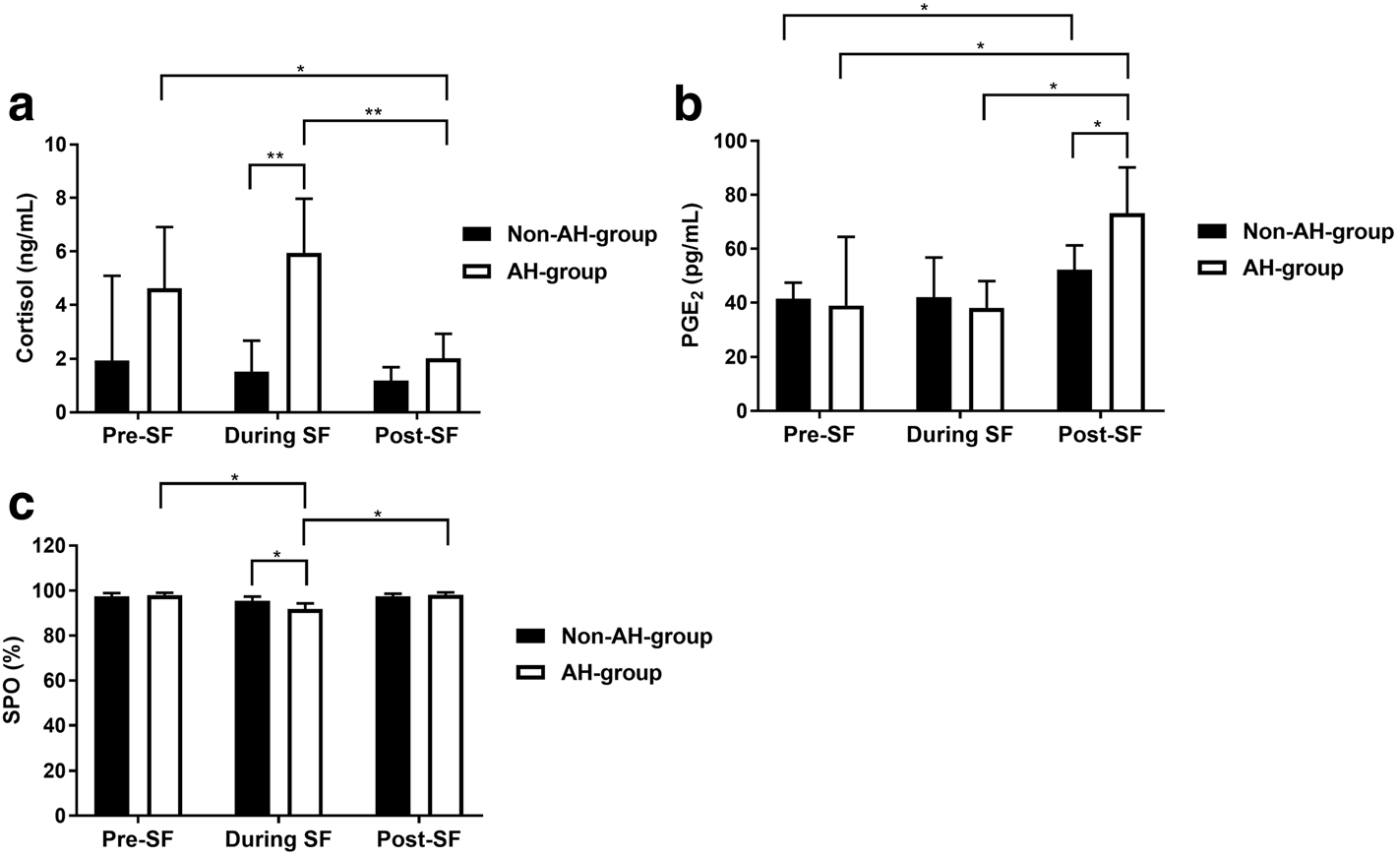
Between and within group comparisons for cortisol (**a**), PGE_2_ (**b**) and SPO (**c**). SF: Simulated flight. Bars indicate SD. *: *p* < 0.05. **: *p* < 0.001

Between group comparison showed that cortisol level was not statistically different in the non-AH-group (1.94 ± 3.15 ng/ml) compared to the AH-group (4.62 ± 2.29 ng/ml) at pre-simulated flight (*p =* 0.062). However, cortisol was significantly higher in AH-group (5.94 ± 2.03 ng/ml) compared with non-AH-group (1.52 ± 1.16 ng/ml) during the simulated flight (*p <* 0.001). No significant difference was found for cortisol in the non-AH-group (1.19 ± 0.50 ng/ml) compared to the AH-group (2.02 ± 0.92 ng/ml) at post-simulated flight (*p =* 0.053) (see Fig. 1a).

Within group comparison in the non-AH-group demonstrated that cortisol did not show any alterations when it was compared between the time points: pre-simulated flight and during simulated flight (*p =* 1.00), during simulated flight and post-simulated flight (*p =* 0.93) or pre-simulated flight and post-simulated flight (*p =* 1.00) (see Fig. 1a).

Within group comparison in the AH-group, cortisol stayed unaltered between pre-simulated flight and during simulated flight (*p =* 0.29) but it showed a significant difference between during simulated flight and post-simulated flight (*p <* 0.001) and between pre-simulated flight and post-simulated flight (*p =* 0.02) (see Fig. 1a).

### PGE_2_ concentration

Between group comparison showed that PGE_2_ was not statistically different in the non-AH-group (41.43 ± 6.03 pg/mL) compared to the AH-group (38.93 ± 25.55 pg/mL) at pre-simulated flight (*p =* 0.82). Non-AH-group (42.13 ± 14.63 pg/mL) compared to the AH-group (38.16 ± 9.85 pg/mL) during the simulated flight did not show any significant difference either (*p =* 0.52). However, PGE_2_ was statistically different in the non-AH-group (52.28 ± 9.02 pg/mL) compared to the AH-group (73.32 ± 16.87 pg/mL) at post-simulated flight (*p =* 0.01) (see Fig. 1b).

Within group comparison in the non-AH-group, demonstrated that PGE_2_ did not show any remarkable alterations when it was compared between the time points of pre-simulated flight and during simulated flight (*p =* 1.00), or during simulated flight and post-simulated flight (*p =* 0.41). However, a significant difference was found between pre-simulated flight and post-simulated flight (*p =* 0.01) (see Fig. 1b).

Within group comparison in the AH-group, showed no statistically significant difference in PGE_2_ levels between pre-simulated flight and during simulated flight (*p =* 1.00). However, a significant difference was found during simulated flight and post-simulated flight (*p =* 0.01) and between pre-simulated flight and post-simulated flight (*p =* 0.04) (see Fig. 1b).

### Saturation pulse oxygen (SPO)

Between group comparison showed that SPO was not statistically different in the non-AH-group (97.43 ± 1.51%) compared to the AH-group (98.00 ± 1.00%) at pre-simulated flight (*p =* 0.49). However, SPO was significantly different in the non-AH-group (95.57 ± 1.81%) compared to the AH-group (91.85 ± 2.48%) during the simulated flight (*p <* 0.001). At post-simulated flight, SPO was not statistically different between the non-AH-group (97.57 ± 1.13%) and the AH-group (98.14 ± 1.06%) (*p =* 0.095) (see Fig. 1c).

Within group comparison in the non-AH-group, indicated that SPO did not show any difference when it was compared between the time points of pre-simulated flight and during simulated flight (*p =* 0.06), during simulated flight and post-simulated flight (*p =* 0.07) or pre-simulated flight and post-simulated flight (*p =* 1.00) (see Fig. 1c).

Within group comparison in the AH-group, SPO was significantly different between pre-simulated flight and during simulated flight (*p <* 0.001), during simulated flight and post-simulated flight (*p <* 0.001), but did not show a difference between pre-simulated flight and post-simulated flight (*p =* 1.00) (see Fig. 1c).

### Pulse rate

**Fig. 2:**
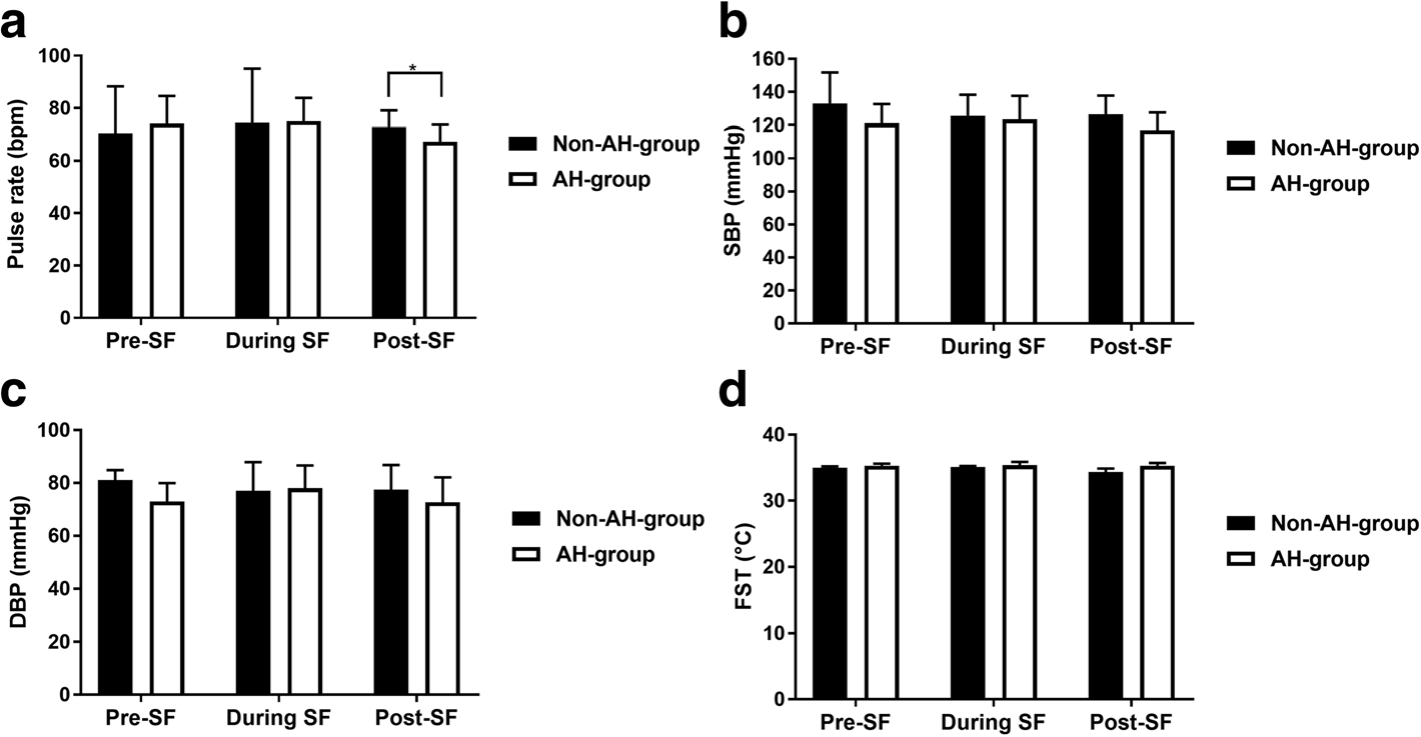
Between and within group comparisons for pulse rate (**a**), SBP (**b**), DBP (**c**) and facial skin temperature (FST) (**d**). SF: Simulated flight. Bars indicate SD.*: *p <* 0.05

Between group comparison showed that the pulse did not show any significant difference in the non-AH-group (70.43 ± 17.85 bpm) compared to the AH-group (74.14 ± 10.51 bpm) at pre-simulated flight (*p =* 0.62). No difference was found in the non-AH-group (74.43 ± 20.64 bpm) compared to the AH-group (75.00 ± 8.91 bpm) during the simulated flight, (*p =* 0.96), or post-simulated flight (*p =* 0.20) in the non-AH-group (72.86 ± 6.23 bpm) compared to the AH-group (67.29 ± 6.40 bpm) (see Fig. 2a).

Within group comparison in the non-AH-group, indicated that the pulse did not show any significant variations between pre-simulated flight and during simulated flight (*p =* 0.74), during simulated flight and post-simulated flight (*p =* 1.00), or pre-simulated flight and post-simulated flight (*p =* 1.00) (see Fig. 2a).

Within group comparison in the AH-group, presented a similar observation, where pulse did not show a significant alteration through assessment time-points: between pre-simulated flight and during simulated flight (*p =* 1.00), during simulated flight and post-simulated flight (*p =* 1.00), or pre-simulated flight and post-simulated flight (*p =* 0.93) (see Fig. 2a).

### Systolic blood pressure (SBP)

Between group comparison showed that SBP was not significantly different in the non-AH-group (133.14 ± 18.69 mmHg) compared to the AH-group (121.14 ± 11.57 mmHg) at pre-simulated flight (*p =* 0.26). This was also the case when SBP was compared between the non-AH-group (125.71 ± 12.58 mmHg) and the AH-group (123.43 ± 14.27 mmHg) during the simulated flight (*p =* 0.77), and also in the non-AH-group (126.57 ± 11.19 mmHg) compared to the AH-group (117.00 ± 10.60 mmHg) at post-simulated flight (*p =* 0.13) (see Fig. 2b).

Within group comparison in the non-AH-group, indicated that SBP did not show any significant variations when the comparisons were performed between pre-simulated flight and during simulated flight (*p =* 0.78), during simulated flight and post-simulated flight (*p =* 1.00), or pre-simulated flight and post-simulated flight (*p =* 1.00) (see Fig. 2b).

Within group comparison in the AH-group, also indicated similar findings. SBP did not vary significantly when comparisons performed between pre-simulated flight and during simulated flight (*p =* 0.50), during simulated flight and post-simulated flight (*p =* 1.00), or pre-simulated flight and post-simulated flight (*p =* 1.00) (see Fig. 2b).

### Diastolic blood pressure (DBP)

Between group comparison showed that DBP was not different in the non-AH-group (81.14 ± 3.72 mmHg) compared to the AH-group (73.00 ± 6.98 mmHg) at pre-simulated flight (*p =* 0.69), nor was it different in the non-AH-group (77.00 ± 10.86 mmHg) compared to the AH-group (78.00 ± 8.66 mmHg) during the simulated flight (*p =* 0.86), or in the non-AH-group (77.43 ± 9.40 mmHg) compared to the AH-group (72.71 ± 9.39 mmHg) at post-simulated flight (*p =* 0.34) (see Fig. 2c).

Within group comparison in the non-AH-group, presented that DBP did not show a variation between the assessments’ time-points: pre-simulated flight versus during simulated flight (*p =* 1.00), during simulated flight and post-simulated flight (*p =* 1.00), or pre-simulated flight and post-simulated flight (*p =* 1.00) (see Fig. 2c).

Within group comparison in the AH-group showed no significant change in DBP when comparisons were performed between pre-simulated flight and during simulated flight (*p =* 0.15), during simulated flight and post-simulated flight (*p =* 0.12), or pre-simulated flight and post-simulated flight (*p =* 1.00) (see Fig. 2c).

### Facial skin temperature

Between group comparison showed that the average facial skin temperature was not statistically different between the non-AH-group (34.97 ± 0.24 °C) and the AH-group (35.31 ± 0.30 °C) at pre-simulated flight (*p =* 0.12). The facial skin temperature did not show any significant difference between the non-AH-group (35.10 ± 0.14 °C) and the AH-group (35.37 ± 0.52 °C) during the simulated flight (*p =* 0.19), or at post-simulated flight (*p =* 0.33), where non-AH-group (34.36 ± 0.53 °C) compared with the AH-group (35.30 ± 0.42 °C), (see Fig. 2d).

Within group comparison in the non-AH-group, indicated that the facial skin temperature did not show any significant difference between pre-simulated flight and during simulated flight (*p =* 1.00), during simulated flight and post-simulated flight (*p =* 1.00), or pre-simulated flight and post-simulated flight (*p =* 1.00) (see Fig. 2d).

Within group comparison in the AH-group, also presented that no significant alteration in the facial skin temperature occurred when it was compared between pre-simulated flight and during simulated flight (*p =* 1.00), during simulated flight and post-simulated flight (*p =* 1.00), or pre-simulated flight and post-simulated flight (*p =* 1.00) (see Fig. 2d).

## Discussion

This study is the first of its kind to simulate the conditions of a real flight in order to allow investigating of possible mechanisms underlying AH. Findings from our simulation study with the pressure chamber suggest that the conditions applied here could resemble those seen during real flight in an airplane. All participants in the AH-group experienced a headache under simulated condition suggesting that the model works. Other features of AH were also in accordance with AH symptoms often reported during a real flight (see Table 2). The real AH intensity is often described as severe (for example 8.8/10 [6]), while it was rated in average as 6.1/10 under present simulation study. It should be noted that 3 out of 7 in the AH-group rated their headache intensity as 3-4/10 and the rest rated it as 7 or above. In the study by Mainardi et al. [6] up to 85% of AH-patients described their pain as severe. Therefore, it is not unlikely that AH also appears with lower pain intensity (15%). Potentially, conditions of the simulated flight such as time, cruising altitude, and speed in the current study have also contributed in some variances in pain intensity when compared with real flights. The duration of our simulated flight was only one hour due to the safety considerations and regulations applied to the use of the pressure chamber. Regardless of flight length, or altitude, AH often lasts less than 30 min [1, 6]. In some cases, a milder second phase of headache could also occur after the severe pain is disappeared and could last for several hours. These patients represent a subgroup of AH [8].

**Table 2:** Clinical characteristics of real-time AH (*n* = 75) compared with simulated AH

Characteristics	Real-time AH [6]	Simulated AH
Number of subjects	75	7
Onset of AH	Mainly at landing	Descending phase
Duration of AH	10-30 min	26 min^a^
Intensity of pain	8.8/10	6.1/10^a^
Quality of pain	Stabbing, pulsating	Stabbing, pulsating
Localization	Fronto-orbital, unilateral	Fronto-orbital, unilateral

^a^Mean calculations of all the subjects in the AH-group (*n* = 7)

### Cortisol

The concentration of cortisol was elevated during the simulated flight for the AH-group, whereas it dropped for all members of the non-AH-group during the stay in the pressure chamber. This is somewhat in accordance to the results from the study by Simeoni et al. [27], who claimed that the salivary cortisol level elevated significantly in their healthy participants in a simulated flight. The increase in the cortisol level for these participants was particularly high, which is believed to be due to the fact that the simulated flight reached an altitude of 7620 m, which induced hypoxia. The participants were divided into two groups. One group had the oxygen masks on during the simulated flight, whereas the other group did not wear any oxygen masks. Considering that in our simulated flight that only reached an altitude of 2438 m, and no one had any mask on, it could be explained as to why the cortisol levels in our healthy participants are not severely affected. It has previously been shown that certain passengers that suffers from AH, have developed anxiety at the time of flight traveling [6]. It has also been proven that people suffering from anxiety have elevated levels of cortisol [28]. Our simulated flight was assisted by safety observers who could immediately stop the simulated flight in the pressure chamber if it was necessary and this was a source of confidence for all participants. However, our participants suffering from AH were aware of the fact that they were about to experience a simulated flight, and thus might have a risk of developing a bout of AH in the chamber, which was reflected on elevated levels of cortisol. In this group, cortisol levels are significantly higher before and during the simulated flight. We do note, however, that cortisol levels in both groups are almost equal after the simulated flight, which might suggest, that both groups are relieved to see that the simulated flight has come to an end. Simeoni et al. [27] has shown that the group wearing oxygen masks in their study only experienced a slight raise in cortisol levels during the simulated flight. It has been proven that headache can be triggered by extended durations of hypoxia. It could be of great interest to see if the use of oxygen masks by passengers suffering from AH would decrease cortisol levels, or prevent bouts of AH during flight.

### PGE_2_

The PGE_2_ levels for both the AH- and the non-AH-groups were almost identical both before and during the simulated flight. However, the PGE_2_ level was found elevated in both groups after the flight, where the increase was higher in the AH-group. This is in accordance with Benedetti et al. [29] who showed that the PGE_2_ level increased significantly, when participants were moved to an altitude of 3500 m [29], where they were exposed to hypoxia at this altitude. All participants in this study were healthy volunteers, who experienced HAH, while situated in an elevated position. HAH can be a risk factor for AH as it was reported in a recent study by Bui et al. [2] . Even though HAH and AH are two separate headaches, hypoxia might be one of the main players in both headaches as a consequence of atmospheric pressure alteration. We therefore speculate that there might be a possible relation between HAH and AH [2]. Our data show that the PGE_2_ level is increased significantly after the simulated flight for the AH-group, which might show a potential link with development of AH. PGE_2_ is known for causing vasodilation in the cerebral arteries and it is thought to play a major role in development of certain headaches [14–18]. Ipekdal et al. [10] demonstrated that triptans cause vasospasm in cerebral arteries and thereby prevent AH if the drug is administered 30 min before the flight travel. It is well-known that vasodilation of the cerebral arteries occurs during a migraine attack and that triptians are effective in acute migraine [30, 31]. At least in part, based on effectiveness of triptans, vasodilation of the cerebral arteries might be one of the potential causes of AH [2, 10]. An interesting finding by Benedetti et al. [29], was that the use of oxygen masks decreased the PGE_2_ level as well as the intensity of the HAH. Due to the low atmospheric pressure in the airplane, the body would absorb less oxygen, where hypoxia can lead to headache [2, 32, 33]. It remains uncertain whether hypoxia is associated with AH or not, but it would be interesting to examine if an increase in cabin oxygen levels or use of oxygen masks can decrease the PGE_2_ level and consequently prevent AH. Another possible way of preventing AH, might be by aid of EP_4_ receptor antagonist, BGC20-1531. It has been shown that elevated levels of PGE_2_ up-regulate EP_4_-receptors, which has been suggested as a target for pain treatment [34]. It might be of great interest to see if BGC20-1531 can be used in the future to prevent AH.

### SPO

The average SPO for the healthy participants was found decreased in both groups during the simulated flight in the pressure chamber. A previous study has shown that the SPO decreases during flight [28]. A total of 84 healthy participants were examined in that study. The SPO decreased on an average from 97% to 95% in 46% of the population during a flight lasting at least for an hour. Despite the fact that our population only consists of 14 participants, our data indicate that the SPO is different for healthy participants compared to participants suffering from AH. The SPO for our healthy participants decreased gradually from 97.57 ± 1.13% to 95.57 ± 1.81%, which is in accordance with the results by Humphreys et al. [28]. The drop was significant for the AH-group, where the saturation decreased from 98.00 ± 1.00% to 91.85 ± 2.48%. The decreased SPO for both groups might be the result of the low atmospheric pressure inside the pressure chamber. It has been shown that the cabin pressure decreases with 8 hPa for every increasing 300 m, until it stabilizes at 846 hPa for the remaining duration of the flight at an altitude of 2500 m [35]. It has been proposed that lower atmospheric pressure can lead to a headache due to hypoxia [11]. As compensation, human body would absorb a lesser amount of oxygen as pressure drops. Our data also indicate that the average SPO was elevated after the participants left the pressure chamber. It remains unclear whether the AH-group is more sensitive to the changes in the atmospheric pressure, and whether or not the low saturation alone can trigger a headache with AH features in this group. If this is the case, it would be interesting to see if an increased oxygen concentration in the cabin could alter the outcome.

### Pulse, SBP and DBP

Our data indicate a gradual increase in pulse for both groups of non-AH-group and AH-group during the simulation trial in the pressure chamber, even though the increase did not reach to a significant change statistically. This finding is in accordance with the study by Gardiner et al. [36] who examined the effect of altitude on blood pressure and pulse in healthy participants. The participants were elevated to an altitude of 1828 m, which caused a decrease in blood pressure and an increase in pulse. Another study by Bian et al. [37] has also measured pulse and blood pressure for participants with HAH during a flight at the altitude of 3500 m, where they showed an increased blood pressure and pulse. However, the increased blood pressure was not significantly correlated with HAH. An increased pulse is a normal physiological compensation in human body, when the atmospheric pressure is low. SBP and BDP for the non-AH-group indicated a decrease during the flight, which to some extent correlates with the results by Gardiner et al. [36]. The SBP and BDP for the AH-group increased gradually during the simulated flight, which was similar to the study by Bian et al. [37]. It is known that healthy participants can compensate changing pressure by lowering the arterial blood pressure [36]. It remains unclear however, if the gradual elevation of blood pressure and pulse frequency would play a role in the development of HAH, and thereby in AH, which requires further studies. The study by Bian et al. [37] indicates that a significant elevated pulse can trigger a bout of HAH. A non-significant increased pulse was observed in our AH-group during the simulated flight, but this gradual increasing has potentially been insufficient to evoke an attack or might be just indicating that elevated pulse might not be a trigger for AH. Furthermore, it has been shown that one patient with AH had a normal pulse and blood pressure, but it is unknown if recordings were performed during the flight [38]. Pulse and blood pressure might be influenced by multiple confounding factors and this point should definitely be considered when interpreting data.

### Facial skin temperature

We measured facial skin temperature around the nose, sinuses and the temporal areas, to record if any alteration would occur. Studies have shown that the body temperature is slightly elevated in higher altitudes [39]. We expected to see skin temperature alterations in the pressure chamber. An insignificant tendency was seen, that the average facial skin temperature was higher in the AH-group compared to the non-AH-group during simulation in the chamber. The study by Drummond et al. [40] has examined the facial temperature among migraine patients and healthy participants. Their results showed that the facial temperature in the orbital region was significantly higher among migraine patients during a migraine attack compared to healthy participants. Vasodilation of the cerebral arteries might be considered as a potential cause of AH and migraine where both headaches respond to triptans [2, 10]. Gradual elevation of facial skin temperature in the AH-group, compared with the non-AH-group in the chamber, could have been triggered by an internal occurrence of a potential vasodilation process. This needs further investigation to identify whether it can be systemic, or regional, peripheral or central or both and to what extent. It should be noted that the participants wore ear protection during their stay in the chamber, which interfered with the proper measurement of the average temperature on facial region. Had the participants been able to remove the earmuffs (not possible due to need for communication/extreme noise), then the average temperature would presumably have been recorded accurately. However, the average temperature between the two groups and between the three time-slots did not show any significant alterations. It is plausible that temperature changes could be relevant. A super sensitive technique or proper preparations might yield a different outcome.

**Fig. 3:**
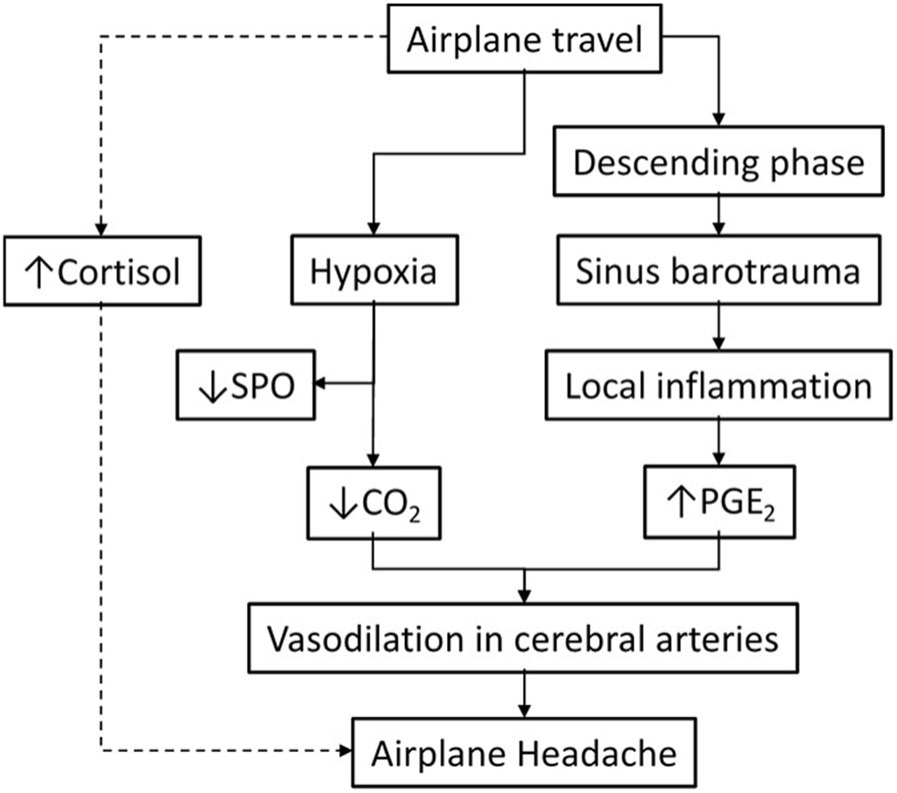
Proposed mechanisms underlying development of AH. Berilgen et al. [7] suggest that AH results from local inflammation caused by sinus barotrauma due to changes in the atmospheric pressure during the landing. The first degree of sinus barotrauma [41] is a short lasting discomfort with almost no anatomical changes in the sinuses and can be considered as a potential player in AH. Most passengers sense pressure changes during landing and gas trapping in the sinuses often occurs that can contribute in sinus barotrauma and a transient local inflammation. Since we identified that PGE_2_, an inflammation mediator, was higher in AH-group and SPO was lower, we suggest that a mild hypoxia may occur during a flight travel that may lead to reflective but perhaps mild hyperventilation. Hyperventilation can result in decreasing levels of carbon dioxide (CO_2_) and elevated blood pH [42]. As a response to the decreased CO_2_, vasodilation may occur [42]. Vasodilation in the cerebral arteries as a reaction to local inflammation or hypoxia can theoretically lead to development of AH. This hypothesis should be examined. Furthermore, interrelationship between anxiety, stress and other environmental and internal subjective factors linked with AH should also be investigated thoroughly to approve or falsify the theoretical model presented here

In summary, several mechanisms have been postulated to explain the alterations seen in AH. Fig. 3 presents proposed mechanisms underlying development of AH as described here based on present findings and other cited studies.

## Conclusion

The present study showed a significant difference in the values of PGE_2_, cortisol and SPO during an AH-attack compared with healthy participants. These biomarkers can attribute to a better understanding of the underlying mechanisms in AH. The pressure chamber succeeded in inducing an AH-like attack in the AH-group, which gives rise to the possibility of using the pressure chamber as a model to simulate AH on the ground and thereby facilitating assessment of other potential biomarkers or further AH investigations in general.
